# Impact of Acute Confusional State in Patients With COVID-19 and a Predictive Score

**DOI:** 10.7759/cureus.18360

**Published:** 2021-09-28

**Authors:** Bhanu Gogia, Deep Pujara, Neeharika Thottempudi, Tamer Ghanayem, Yousaf Ajam, Ayush Singh, Alok Dabi, Shekhar Patil, Kyra Curtis, Xiang Fang, Kamakshi Patel, Anish Bhardwaj, Prashant Rai

**Affiliations:** 1 Neurology/Vascular Neurology, Beth Israel Deaconess Medical Center, Harvard Medical School, Boston, USA; 2 Neurology, Case Western Reserve University, Cleveland, USA; 3 Neurology, University of Texas Medical Branch at Galveston, Galveston, USA; 4 Neurology, Vanderbilt University Medical Center, Nashville, USA; 5 Neurology, Emory University School of Medicine, Atlanta, USA; 6 Internal Medicine, Northeast Internal Medical Associates, Fort Wayne, USA

**Keywords:** receiver operating characteristic (roc) analysis, retrospective cohort, predictive score, acute confusional state, covid-19

## Abstract

Background: Acute confusional state (ACS) in COVID-19 is shown to be associated with poor clinical outcomes.

Methods: We assessed the impact of ACS - defined as a documented deterioration of mental status from baseline on the alertness and orientation to time, place, and person - on inpatient mortality and the need for intensive care unit (ICU) transfer in inpatient admissions with active COVID-19 infection in a single-center retrospective cohort of inpatient admissions from a designated COVID-19 tertiary care center using an electronic health record system. Furthermore, we developed and validated a neurological history and symptom-based predictive score of developing ACS.

Results: Thirty seven out of 245 (15%) patients demonstrated ACS. Nineteen (51%) patients had multifactorial ACS, followed by 11 (30%) patients because of hypoxemia. ACS patients were significantly older (80 [70-85] years vs 50.5 [38-69] years, p < 0.001) and demonstrated more frequent history of dementia (43% vs 9%, p < 0.001) and epilepsy (16% vs 2%, p = 0.001). ACS patients observed significantly higher in-hospital mortality (45.9% vs 1.9%, aOR [adjusted odds ratio]: 15.7, 95% CI = 3.6-68.0, p < 0.001) and need for ICU transfer (64.9% vs 35.1%, aOR: 2.7, 95% CI = 1.2-6.1, p = 0.015). In patients who survived hospitalization, ACS was associated with longer hospital stay (6 [3.5-10.5] days vs 3 [2-7] day, p = 0.012) and numerically longer ICU stay (6 [4-10] days vs 3 [2-6] days, p = 0.078). A score to predict ACS demonstrated 75.68% sensitivity and 81.73% specificity at a cutoff of ≥3.

Conclusion: A high prevalence of ACS was found in patients with COVID-19 in our study cohort. Patients with ACS demonstrated increased mortality and need for ICU care. An internally validated score to predict ACS demonstrated high sensitivity and specificity in our cohort.

## Introduction

The COVID-19 pandemic that initially presented as a cluster of unexplained pneumonia cases in Wuhan, China, in December 2019 [1] and rapidly spread across the world has reached more than 170 million confirmed cases as of June 1, 2021 [2], with South-East Asia and Americas leading in the total new cases (May 24, 2021-May 31, 2021). Strict confinement protocols combined with enhanced vaccination efforts have led to some degree of control in disease transmission in the United States, Europe, and Australia; however, failure to curb the spread in South-East Asia and South America ascertains continued propagation of the disease resulting in high mortality [2]. While further understanding of the disease pathophysiology has helped to decrease mortality due to cytokine storms in the initial phase of the disease, the lack of availability of highly effective therapies to control viral propagation means overall mortality remains high. Propagation of newer strains has resulted in the so-called second wave of transmission, causing havoc on countries with limited resources like India. Until better control and effective vaccination measures are available worldwide, better strategies for risk stratification and prognostication, as well as early identification of patients who may need enhanced care, may help reduce the care burden and improve outcomes [3].

While very few studies have identified potential direct nervous system involvement in COVID-19 [4], neurological symptoms due to multisystem involvement are frequent in patients presenting with severe acute respiratory syndrome coronavirus 2 (SARS-CoV-2) [5-9]. Acute confusional state (ACS) is one such symptom that has been shown to be frequently present in COVID-19 patients. ACS is usually a harbinger of catastrophic events and requires extensive workup and management to prevent poor outcomes [5-10]. However, the overall impact of ACS in COVID-19 has not been quantified well.

We aimed to evaluate the prognostic utility of ACS at presentation or during hospitalization in predicting the in-hospital mortality, need for intensive care support, and hospital length of stay in patients surviving the disease. Furthermore, we aimed to identify predictors of ACS in patients presenting with COVID-19 and create a differential scoring system using these predictors, which can provide a reasonable risk classification of developing ACS during the course of the disease.

## Materials and methods

Study design and population

A retrospective review of the electronic health record system at the University of Texas Medical Branch at Galveston, Texas, was conducted to identify consecutive inpatient admissions with a confirmed diagnosis of COVID-19 between March 1, 2020, and July 13, 2020. The hospital serves as a tertiary care center for five to six cities in the southeast Texas region, with an average population of 415,000. Patients who presented with a confirmed diagnosis of COVID-19 but were not admitted to the hospital were excluded from the study. ACS was defined as a documented deterioration of mental status from baseline on the alertness and orientation to time, place, and person. Patients were stratified based on the presence or absence of ACS either at the time of admission or during the hospital stay.

The study was approved by the University of Texas Medical Branch at Galveston Institutional Review Board, and the approval number was 20-0142. A waiver of informed consent was granted by the Institutional Review Board as the study was a retrospective review of already collected health records and possessed no more than minimal risk to the study participants.

COVID-19 diagnosis and management protocols

All patients presenting to the emergency room were initially screened for COVID-19-related symptoms. For all symptomatic patients, a nasopharyngeal swab sample was obtained and processed using real-time reverse transcriptase-polymerase chain reaction (PCR) test [11]. Patients confirmed to have an infection with SARS-CoV-2 were transferred to a dedicated COVID unit. An institutional protocol was implemented to standardize the management of COVID-19 patients, with the provision of supplemental oxygen if PaO_2_ < 93% on room air, monitoring in an intensive care unit (ICU) if the patient requires multi-organ support and intubation, and mechanical ventilation provided if patients develop respiratory failure and hypoxia despite high-flow supplemental oxygen. Participation in ongoing clinical trials was also documented.

Study variables

Health records were examined to collect and ascertain demographic information, including age at presentation, sex, race, and body mass index (BMI). History of conditions including hypertension, diabetes mellitus, dyslipidemia, obesity, coronary artery disease, and chronic kidney disease was collected to identify the burden of comorbidity. The focus of the study was information regarding the history of neurological illnesses including prior ischemic or hemorrhagic stroke and neurological symptoms at presentation and during hospitalization including agitation and delirium, dizziness, anosmia, dysgeusia, seizures, muscle pain, fatigue, dysarthria, and diplopia with the duration of symptoms.

The primary outcome was inhospital mortality. ICU transfer was the secondary outcome. Evaluation of overall hospital length of stay and ICU length of stay was also completed.

Statistical analysis

STATA 15 software (StataCorp LLC, College Station, Texas) was used for all statistical analyses. All p-values were two-sided, and p < 0.05 was considered statistically significant.

Baseline characteristics and outcomes were assessed and compared between patients who presented with and without ACS, either at the time of admission or during hospitalization. Univariate comparisons were made using Pearson’s chi-squared test [12] or Fisher’s exact test [13] for categorical variables and using Student’s t-test [14] or Mann-Whitney U test [15] for continuous variables, as appropriate.

The effect of ACS on in-hospital mortality and ICU transfer using a multivariable logistic regression model was assessed. Age, sex, and all variables with a univariate p-value of <0.1 were included in the initial models. A backward stepwise method [16] of variable selection was employed, excluding all variables with p < 0.1 to ensure a parsimonious model.

We also evaluated predictors of ACS using a multivariable logistic regression model obtained by the backward stepwise variable selection, as described above. A scoring system using predictors of ACS was developed using scores based on their odds ratio (OR) from the multivariable model. The final score was created by summing up all the scores for individual variables. Using received operating characteristics (ROC) analysis, the optimal differential point was identified by maximizing Youden’s J index [17]. Using this point as a cutoff, patients were classified into low and high probability of ACS. Sensitivity and specificity were calculated and reported, with corresponding area under the curve (AUC) statistics. Internal validation using 1000 study cohorts created by stratified substitution with replacement (bootstrapping) was conducted to ensure the homogeneity of estimates obtained. We reported the mean AUC statistics as well as sensitivity and specificity at an optimized cutoff for the bootstrapping replications in an effort to internally validate the score.

## Results

Patient population

A total of 245 patients were admitted to the University of Texas Medical Branch at Galveston, Texas, with a laboratory-confirmed SAS-CoV-2 infection between March 01, 2020, and July 13, 2020. Median (IQR) age was 55 (39, 75) years, and 124 (51%) patients were females; 68 (28%) patients demonstrated history of at least one neurological disease, whereas 112 (46%) patients presented with at least one neurological symptom.

**Figure 1:**
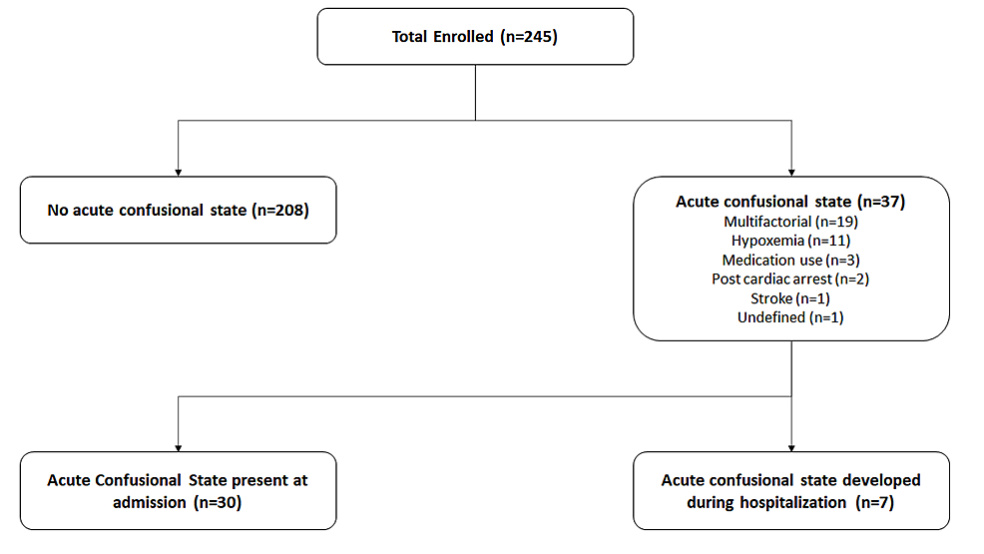
Flow diagram of the study cohort

Thirty-seven (15%) patients demonstrated ACS, either present at admission or developed during hospitalization. Thirty (81%) patients had ACS present at the time of admission with a history of altered mental status on a median (IQR) of 1 (1-2) day. Seven patients developed ACS during their hospital stay, on a median (IQR) of 5.5 (3-8) days after admission. Nineteen (51%) patients had multifactorial etiology of ACS, followed by 11 (30%) patients with hypoxemia resulting in ACS. Three (8%) patients had ACS due to medication use at baseline, whereas three (8%) patients had vascular etiology (two post-cardiac arrest encephalopathy and one stroke). Etiology could not be ascertained in one patient due to rapid clinical decline and death soon after admission. Figure 1 demonstrates the flow chart of patients presenting with COVID-19, stratified based on the development of ACS either at the time of admission or after hospitalization.

Comparison of baseline characteristics in patients with and without acute confusional state

A comparison of baseline clinical and biochemical characteristics between patients who did and did not exhibit ACS is provided in Table 1. Patients who demonstrated ACS were significantly older (ACS: 80 [70-85] years vs no ACS: 50.5 [38-69] years, p < 0.001). The distribution of gender was similar between the two groups (Males - ACS: 19 [51.4%] vs no ACS: 102 [49.0%], p = 0.80). Patients with ACS demonstrated significantly higher comorbidities, including hypertension, congestive heart failure, obesity, coronary artery disease, and chronic kidney disease.

**Table 1:** Baseline clinical and biochemical characteristics in patients presenting with COVID-19 stratified by the presence or absence of acute confusional state

Baseline Clinical and Biochemical Characteristics		Overall	Acute Confusional State (ACS) Absent	Acute Confusional State (ACS) Present	p-Value	Test
		N = 245	N = 208	N = 37		
Age (years)		55 (39-75)	50.5 (38-69)	80 (70-85)	<0.001	Wilcoxon rank-sum
Sex	Female	124 (50.6%)	106 (51.0%)	18 (48.6%)	0.8	Pearson's chi-squared
	Male	121 (49.4%)	102 (49.0%)	19 (51.4%)		
Race	African-American	46 (18.8%)	40 (19.2%)	6 (16.2%)	0.27	Fisher's exact
	Asian	2 (0.8%)	2 (1.0%)	0 (0.0%)		
	Caucasian	106 (43.3%)	84 (40.4%)	22 (59.5%)		
	Hispanic/Latino	89 (36.3%)	80 (38.5%)	9 (24.3%)		
	Native American	2 (0.8%)	2 (1.0%)	0 (0.0%)		
History of Non-neurological Diseases	Hypertension	149 (61.1%)	117 (56.5%)	32 (86.5%)	<0.001	Pearson's chi-squared
	Diabetes mellitus	84 (34.3%)	69 (33.2%)	15 (40.5%)	0.38	Pearson's chi-squared
	Dyslipidemia	93 (38.3%)	75 (36.4%)	18 (48.6%)	0.16	Pearson's chi-squared
	Congestive heart failure	50 (20.4%)	32 (15.4%)	18 (48.7%)	<0.001	Pearson's chi-squared
	Obesity	129 (54.0%)	119 (58.6%)	10 (27.8%)	<0.001	Pearson's chi-squared
	Coronary artery disease	54 (22.0%)	36 (17.3%)	18 (48.6%)	<0.001	Pearson's chi-squared
	Chronic kidney disease	44 (18.0%)	27 (13.0%)	17 (45.9%)	<0.001	Pearson's chi-squared
History of neurological disorders	Any neurological disorder	68 (27.8%)	44 (21.2%)	24 (64.9%)	<0.001	Pearson's chi-squared
	Demyelinating disorder	2 (0.8%)	2 (1.0%)	0 (0.0%)	1	Fisher's exact
	Neurocognitive disorder	35 (14.3%)	19 (9.1%)	16 (43.2%)	<0.001	Pearson's chi-squared
	Epilepsy	10 (4.1%)	4 (1.9%)	6 (16.2%)	0.001	Fisher's exact
	Migraine/headache syndrome	8 (3.3%)	7 (3.4%)	1 (2.7%)	1	Fisher's exact
	Movement disorders	8 (3.3%)	4 (1.9%)	4 (10.8%)	0.02	Fisher's exact
	Sensory polyneuropathy	17 (6.9%)	10 (4.8%)	7 (18.9%)	0.002	Pearson's chi-squared
	Neuromuscular disorder	4 (1.6%)	3 (1.4%)	1 (2.7%)	0.48	Fisher's exact
	Spinal cord disorders	3 (1.2%)	2 (1.0%)	1 (2.7%)	0.39	Fisher's exact
	Stroke	31 (12.9%)	17 (8.3%)	14 (40.0%)	<0.001	Pearson's chi-squared
Non-neurological symptoms	Fever	158 (64.8%)	135 (64.9%)	23 (63.9%)	0.91	Pearson's chi-squared
	Cough	176 (72.4%)	152 (73.4%)	24 (66.7%)	0.4	Pearson's chi-squared
	Anorexia	96 (39.5%)	74 (35.7%)	22 (61.1%)	0.004	Pearson's chi-squared
	Diarrhea	57 (23.6%)	53 (25.7%)	4 (11.1%)	0.058	Fisher's exact
	Headache	64 (26.2%)	58 (28.0%)	6 (16.2%)	0.13	Pearson's chi-squared
Neurological symptoms	Agitation and delirium	33 (13.5%)	11 (5.3%)	22 (59.5%)	<0.001	Pearson's chi-squared
	Dizziness	27 (11.0%)	21 (10.1%)	6 (16.2%)	0.27	Pearson's chi-squared
	Anosmia	37 (16.2%)	32 (16.4%)	5 (14.7%)	0.8	Pearson's chi-squared
	Dysgeusia	53 (23.1%)	47 (24.1%)	6 (17.6%)	0.41	Pearson's chi-squared
	Seizure	4 (1.6%)	2 (1.0%)	2 (5.4%)	0.11	Fisher's exact
	Myalgia	95 (39.4%)	79 (38.5%)	16 (44.4%)	0.5	Pearson's chi-squared
	Fatigue	166 (68.3%)	141 (68.1%)	25 (69.4%)	0.87	Pearson's chi-squared
	Dysarthria	2 (0.8%)	2 (1.0%)	0 (0.0%)	1	Fisher's exact
	Diplopia	1 (0.4%)	1 (0.5%)	0 (0.0%)	1	Fisher's exact
	Ischemic stroke (new onset)	1 (0.4%)	1 (0.5%)	0 (0.0%)	1	Fisher's exact
Neurology consult obtained		10 (4.1%)	3 (1.4%)	7 (18.9%)	<0.001	Fisher's exact
Neuroimaging obtained	Any neuroimaging obtained	18 (13.4%)	15 (12.4%)	3 (23.1%)	0.38	Fisher's exact
	CT obtained	17 (6.9%)	14 (6.7%)	3 (8.1%)	0.73	Fisher's exact
	MRI obtained	4 (1.6%)	1 (0.5%)	3 (8.1%)	0.011	Fisher's exact
Biochemical characteristics at the time of admission	Total leukocyte count (x10^3^/μL)	7 (5-8.98)	6.74 (4.99-8.81)	7.86 (5.75-11.39)	0.072	Wilcoxon rank-sum
	Absolute neutrophil count (x10^3^/μL)	5.1 (3.57-8.56)	5 (3.52-7.4)	7.05 (4.04-11.785)	0.029	Wilcoxon rank-sum
	Absolute lymphocyte count (x10^3^/μL)	1.075 (0.785-1.65)	1.055 (0.785-1.625)	1.125 (0.765-1.78)	0.53	Wilcoxon rank-sum
	Platelet count (x10^3^/μL)	200 (156-264)	198 (156-258)	216 (159-293)	0.51	Wilcoxon rank-sum
	Serum C-reactive protein (mg/dL)	7.85 (2.6-13.3)	7.8 (2.41-12.9)	9.7 (3.8-18.9)	0.29	Wilcoxon rank-sum
	Erythrocyte sedimentation rate (mm/hour)	36 (15-58)	39 (13.5-62)	36 (21-52)	0.97	Wilcoxon rank-sum
	Serum d-dimer (μg/mL)	0.8 (0.47-1.57)	0.705 (0.43-1.35)	1.312 (0.785-2.885)	0.002	Wilcoxon rank-sum
	Serum creatine kinase (units/L)	98.5 (47-243)	78 (45-204)	160 (87-355)	0.073	Wilcoxon rank-sum
	Serum lactate dehydrogenase (units/L)	724 (521-932)	719.5 (522-899)	732 (517-1012)	0.92	Wilcoxon rank-sum
	Serum alanine aminotransferase (units/L)	31 (20-51)	35 (22-52)	22.5 (14.5-34)	0.006	Wilcoxon rank-sum
	Serum aspartate aminotransferase (units/L)	47 (36-67)	47 (36-67)	42.5 (31.5-70.5)	0.33	Wilcoxon rank-sum
	Blood urea nitrogen (mg/dL)	16 (11-24.5)	15 (10-22)	44 (24-58)	<0.001	Wilcoxon rank-sum
	Serum creatinine (mg/dL)	0.86 (0.65-1.24)	0.81 (0.63-1.07)	1.48 (0.99-3.43)	<0.001	Wilcoxon rank-sum

They also demonstrated more frequent history of at least one neurological diagnosis (ACS: 24 [64.9%] vs no ACS: 44 [21.2%], p < 0.001), including history of dementia (ACS: 16 [43.2%] vs no ACS: 19 [(9.1%], p < 0.001}, epilepsy (ACS: 6 [16.2%] vs no ACS: 4 [1.9%], p = 0.001), movement disorders (ACS: 4 [10.8% vs no ACS: 4 [1.9%], p = 0.02), and stroke (ACS: 14 [40%] vs no ACS: 17 [8.3%], p < 0.001) more frequently. These patients also demonstrated significant differences in symptoms including more frequent anorexia (ACS: 22 [61.1%] vs no ACS: 74 [35.7%], p = 0.004), agitation, and delirium (ACS: 22 [59.5%] vs no ACS: 11 [5.3%], p < 0.001).

Higher comorbidity burden in these patients was also reflected in the biochemical abnormalities such as increased blood urea nitrogen (BUN) (ACS: 44 [24-58] mg/dL vs no ACS: 15 [10-22] mg/dL, p < 0.001), serum creatinine (ACS: 1.48 [0.99-3.43] mg/dL vs no ACS: 0.81 [0.63-1.07] mg/dL, p < 0.001), and higher absolute neutrophil count (ACS: 7.05 [4.04-11.79] x 103/μL vs no ACS: 5 [3.52-7.4] x 103/μL, p = 0.029).

Outcomes in patients with acute confusional state

Patients demonstrating ACS observed significantly higher in-hospital mortality (ACS: 17 [45.9%] vs no ACS: 4 [1.9%], p < 0.001) and need for ICU transfer (ACS: 24 [64.9%] vs no ACS: 73 [35.1%], p < 0.001). In patients who survived hospitalization, ACS was associated with longer hospital stay (ACS: 6 [3.5-10.5] vs no ACS: 3 [2-7], p = 0.012) and a trend toward longer ICU stay (ACS: 6 [4-10] vs no ACS: 3 [2-6] days, p = 0.078). Further details regarding outcomes are provided in Table 2.

**Table 2:** Outcomes in patients presenting with COVID-19 stratified by presence or absence of acute confusional state

	Overall	Acute Confusional State (ACS) Absent	Acute Confusional State (ACS) Present	p-Value	Test
	N = 245	N = 208	N = 37		
Mortality	21 (8.6%)	4 (1.9%)	17 (45.9%)	<0.001	Fisher’s exact
ICU transfer	97 (39.6%)	73 (35.1%)	24 (64.9%)	<0.001	Pearson's chi-squared
Hospital length of stay (days) (including in-hospital deaths)	4 (2-8)	4 (2-7)	8 (3-14.5)	0.002	Wilcoxon rank-sum
ICU length of stay (days) (including in-hospital deaths)	3.5 (2-8)	3 (2-7)	6 (1-9)	0.61	Wilcoxon rank-sum
Hospital length of stay (days) (excluding in-hospital deaths)	4 (2-7)	3 (2-7)	6 (3.5-10.5)	0.012	Wilcoxon rank-sum
ICU length of stay (days) (excluding in-hospital deaths)	4 (2-6)	3 (2-6)	6 (4-10)	0.078	Wilcoxon rank-sum

In multivariable logistic regression models, ACS was an independent predictor of in-hospital mortality (aOR: 15.7, 95% CI = 3.6-68.0, p < 0.001) and need for ICU transfer (aOR: 8.7, 95% CI = 2.4-32.1, p = 0.001).

Predictors of acute confusional state and development of predictive scoring system

Age of the patient (age 65-80 years - aOR: 5.73, 95% CI = 1.18-27.87, p = 0.03; age > 80 years - aOR: 22.06, 95% CI = 3.98-122.25, p < 0.001) along with history of epilepsy (aOR: 9.77, 95% CI = 1.99-48.00, p = 0.005), dementia (aOR: 2.91, 95% CI = 1.03-8.20, p = 0.04) and sensory neuropathy (aOR: 5.11, 95% CI = 1.26-20.60, p = 0.022) were identified as independent predictors of developing ACS in patients with acute COVID-19 infection requiring hospitalization. Using the strength of association with ACS, a relative score was assigned to each predictor, and a cumulative scoring system was created to predict ACS. Detailed information regarding the independent predictors of ACS and the scores assigned is provided in Table 3.

**Table 3:** Predictors of acute confusional state and corresponding predictive score assignment

		Odds Ratio	95% CI	p-Value	Score Assignment
Age	<65 years	Reference			0
	65-80 years	5.73	1.18-27.87	0.03	2
	>80 years	22.06	3.98-122.25	0.0004	7
History of epilepsy	No	Reference			0
	Yes	9.77	1.99-48.00	0.005	3
History of dementia	No	Reference			0
	Yes	2.908	1.03-8.20	0.044	1
History of sensory polyneuropathy	No	Reference			0
	Yes	5.106	1.26-20.61	0.022	2

**Figure 2:**
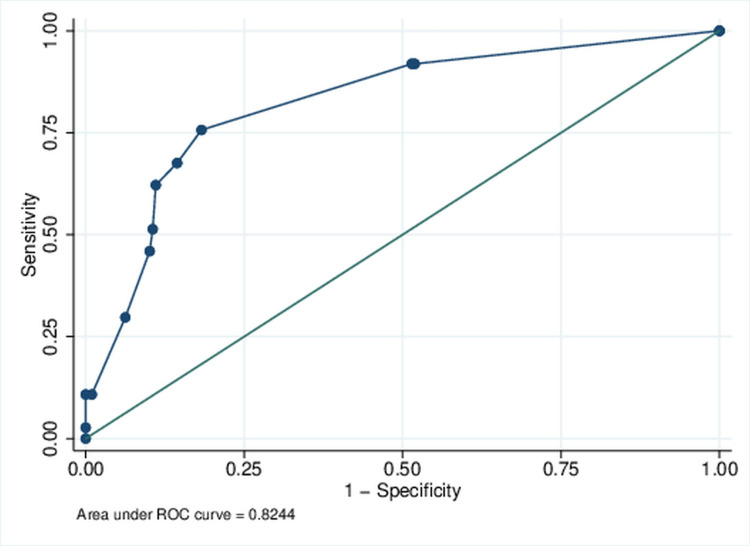
Receiver operating characteristics (ROC) curve for predictive score (continuous) for the acute confusional state

The median score for the included study population was 2 (IQR: 0-3, range: 0-12). Nonparametric ROC analysis demonstrated C-statistics of 0.824, suggesting a high predictive ability of the scoring system in differentiating patients who did and did not develop ACS (Figure 2).

**Figure 3:**
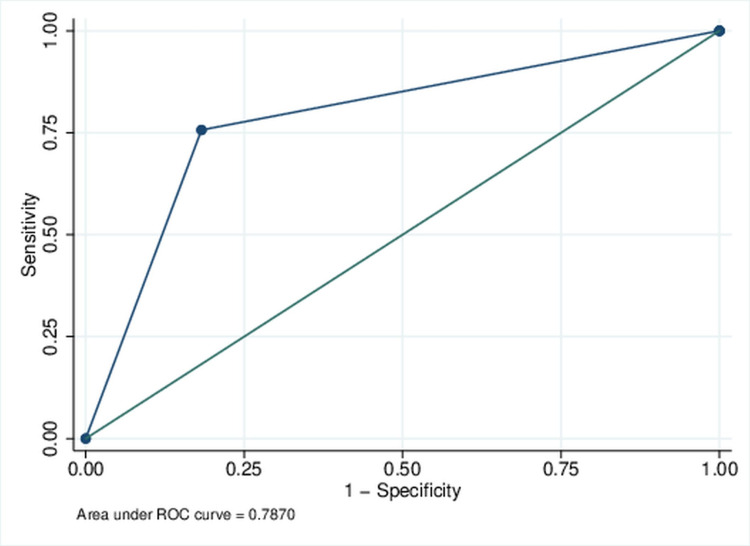
Receiver operating characteristics (ROC) curve for predictive score (dichotomized) for the acute confusional state

Maximal Youden’s J index was 0.782 and was observed at the score cutoff of ≥3. A cutoff of ≥3 identified 66 individuals with high-risk scores (≥3), of which 28 (42.4%) developed an ACS. Of 179 patients with a score of <3, only nine (5%) developed ACS. Sensitivity and specificity at the score cutoff of ≥3 were 75.68% and 81.73%, respectively (Figure 3).

The internal validation of the developed score was executed on 1000 datasets created by stratified sampling with replacement (bootstrapping) from the study population. The mean AUC statistics for the ROC analysis across 1000 datasets was 0.824. Median (IQR) was 0.826 (0.800-0.849). Similarly, in these 1000 datasets, mean sensitivity and specificity were 72.71% and 83.18%, respectively.

## Discussion

Our results demonstrated that the presence of ACS at admission or during hospitalization was associated with poorer outcomes overall, with these higher mortalities and increased need for intensive care support. Among those who survived the hospitalization, the length of hospital stay was significantly prolonged. To our knowledge, this is a rare study to quantify the impact of the association of ACS in COVID-19. The etiology of ACS in COVID-19 is thought to be multifactorial with respiratory failure due to primary disease, co-infections, metabolic impairment due to effect on other organs, and true neurological impairment, all considered to be the underlying causes [5,17-19]. Most of the cases presented to our hospital demonstrated respiratory failure or toxic-metabolic etiology of ACS, with a limited number of cases presenting with vascular etiology. Patients with ACS had higher BUN indicating the toxic-metabolic encephalopathy as an underlying etiology in a significant proportion of patients. History of neurological diseases at baseline was also associated with increased odds of ACS in COVID-19. In line with prior reports demonstrating limited primary involvement of neurological tissue in COVID-19 infections, we did not find any case of neurological involvement due to direct SARS-CoV-2 infection in the brain or spinal cord [20]. The frequency of ischemic strokes was particularly low in our study cohort as compared to prior reports [21,22]. Given that multi-organ involvement was seen in most of our patients, it is important to consider ACS as a systemic marker rather than just one of the neurological symptoms.

A significant proportion of cases presented to our hospital included nursing home residents, thus presenting with more frequent neurological disease history including dementia, epilepsy, stroke, and movement disorders. Interestingly, these patients with prior neurological diseases were also prone to develop ACS more frequently than patients without a history of prior neurological diseases. A multitude of factors including poor baseline function, impaired nutritional status, increased likelihood of infections, and older age can explain the higher frequency of impaired mentation in these patients.

The presence of ACS was associated with significantly higher in-hospital mortality in our patient population. A similar association was found between encephalopathy and mortality in COVID-19 patients in a recent study [23]. While there are several studies that presented neurological manifestations of COVID-19 [5,6,8,9], a handful of studies suggested worse outcomes with neurological comorbidities [18,24-26]. This study signifies the importance of the use of ACS in predicting overall prognosis, and it will be interesting to see if this finding can also be observed and replicated in other studies.

Regarding acuity of the patients and needing ICU level of care either at the time of admission or ICU transfer during the admission, per our hospital protocol, the current criteria for ICU admission include increasing oxygen requirement, oxygen saturation below 93% on room air, and chest x-ray (CXR) with infiltrates suggestive of COVID-19 pneumonia. From the results demonstrated here, patients with ACS had an increased need for ICU level of care either with more direct admissions to ICU or ICU transfers from the ward. This is an important observation as it can give an early insight into the COVID-19 patients who may need higher levels of care.

To address this vital issue, we proposed a novel scoring system that can help to identify this set of patients earlier in the course. Furthermore, as the score is based on neurological history and the age of the patient, it has the added advantage of the ease of use at the bedside. The utility of such scoring systems is well documented in the ICU, where sequential organ failure assessment (SOFA) [27] and acute physiology and chronic health evaluation II (APACHE II) [28] scores are used routinely to identify patients at a higher risk of mortality. It will be important to see if our proposed score can be used in early decision-making that can lead to improvement in the prognosis of COVID-19 patients with relation to mortality and hospital length of stay. The applicability of this score in the settings with limited medical oversight (i.e., nursing homes) may be of particular interest as COVID-19-positive patients who are at higher risk of ACS can be either transferred early to a hospital for a higher level of care or at least be provided increased monitor for deterioration. This could also help triage patients for in-hospital care in countries with limited resources that are currently bearing the brunt of the COVID-19 pandemic with high ensuing mortality.

In congruence with the other studies [23,29], our patients with ACS had an overall longer hospital length of stay as compared to patients with no ACS. So ACS in COVID-19 is associated with poorer prognosis overall with increased mortality and hospital length of stay. Like Garg et al. [18], ACS demonstrated a trend toward a significant increase in ICU length of stay in our study. It is plausible that once the patient is under intensive care, a heightened level of care may decrease the overall impact of ACS. This further indicates the importance of recognizing the ACS as an early indicator of COVID-19 severity.

In the long term, it would be valuable to see how the patients are after the hospital discharge and if ACS has any different long-term complications in COVID-19 as compared to patients with no ACS. Further studies especially in recovered COVID-19 patients after hospital discharge are warranted to look into this. This will be valuable in terms of patient disposition at the time of discharge.

The strengths of our study include the large sample size obtained during the peak of the COVID-19 pandemic, ascertaining the COVID-19 disease with standardized tests, uniform data collection, and limited missing data points. Our study findings are limited by the retrospective nature of the analysis, single-center cohort, evolving diagnostic and treatment paradigms and protocols during the study, and the presence of multiple ongoing blinded clinical trials, which may have confounded the study.

## Conclusions

ACS was associated with poorer outcomes and prognosis in COVID-19 patients in our study. Patients with ACS demonstrated increased odds of in-hospital mortality, need for intensive care, and prolonged length of hospital stay. Advanced age and prior history of neurological diseases were independent predictors of ACS. ACS can play a role as a key indicator for early recognition of patients requiring higher levels of care, which has led to our proposal of a predictive scoring system. Further exploration of the association of ACS with poor outcomes and external validation of the scoring system in COVID-19 is warranted.
